# Revisiting the valence stability and preparation of perovskite structure type oxides ABO_3_ with the use of Madelung electrostatic potential energy and lattice site potential

**DOI:** 10.1039/d1ra01979a

**Published:** 2021-06-10

**Authors:** Masahiro Yoshimura, Kripasindhu Sardar

**Affiliations:** Tokyo Institute of Technology Tokyo Japan yoshimur@ncku.edu.tw; Department of Material Science and Engineering, National Cheng Kung University Tainan 701 Taiwan

## Abstract

Valence stability of aliovalent ions is mostly correlated with lattice site potential in ionic crystals. Madelung electrostatic potential is obtained by adding all the lattice site potentials for all the ions present in a crystal structure. Therefore, valence stability and the stability of a crystal structure can be better understood with consideration of both the lattice site potential and Madelung electrostatic potential. This was first demonstrated more than four decades ago by one of the present authors. We revisit this situation by using re-calculated lattice site potential and Madelung electrostatic potential for perovskite structure type ABO_3_ compounds using a new computer program VESTA. We show that the formation of a perovskite structure type compound with the general formula ABO_3_ (where A and B are cations and O is an oxide ion) becomes energetically favorable when it has a higher Madelung electrostatic potential than the combined Madelung electrostatic potential of parent binary compounds AO and B_2_O_3_ or BO_2_. It is further shown that strong lattice site potential results in stability of high valence or high valence ions can be stabilized in a lattice site with strong lattice-site potential. It further follows that certain ions experience maximum lattice site potential at the B ion lattice site of the perovskite structure when compared to other structures such as fluorite BO_2_, rutile BO_2_ and corundum B_2_O_3_. Therefore, (i) the stability of an ion with a high (and uncommon) valence state at the B site being higher than that at the A site, (ii) occurrence of point defects at A or O sites with weak lattice site potentials, respectively and (iii) instability of perovskite A^4+^B^2+^O_3_, and A^5+^B^1+^O_3_ compounds, respectively can be rationalized by lattice site potential and Madelung electrostatic potential analysis.

## Abstract 2

The stability of perovskite structure type oxides and higth valence state of cations, respectively may be better understood by considering lattice site potential and Madelung electrostatic potential energy.

## Graphical abstract

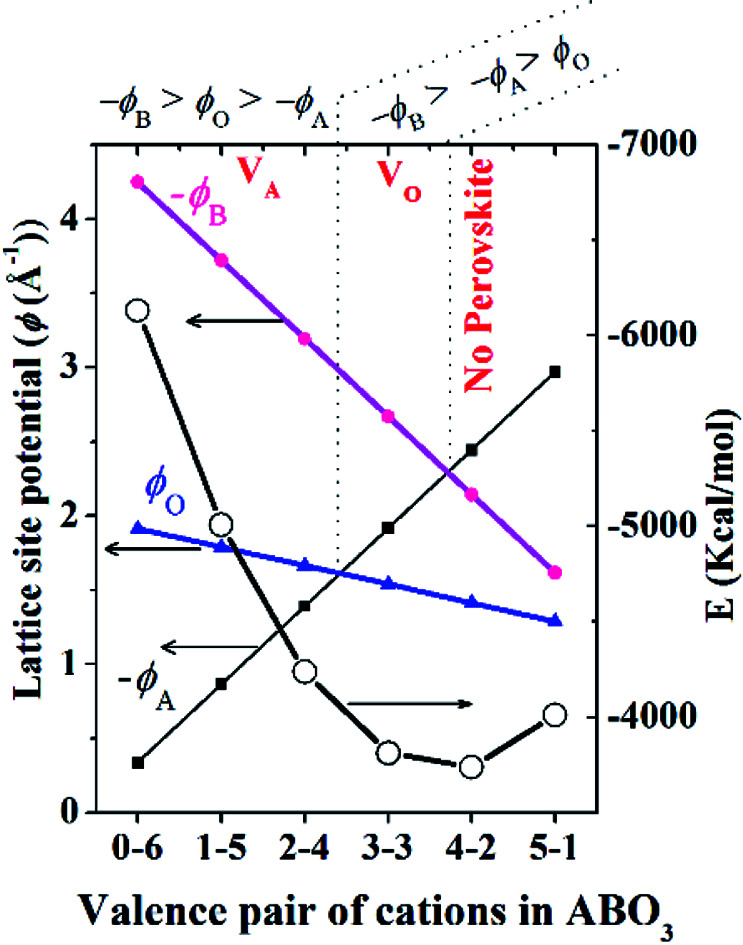

## Introduction

An enormous number of solids show crystallographic structural similarity to the mineral perovskite (CaTiO_3_) and have the general formula ABX_3_. Metal oxides with chemical formula ABO_3_ are a subclass of this large family of compounds. For many years, perovskite oxides have been studied extensively in the literature of chemistry, physics and geology for a large variety of solid state phenomena associated with electric, optical, thermal and magnetic properties. A large number of oxides with perovskite type structures are possible to synthesise in a laboratory for applications in vast areas spanning from electronics to catalysis. It is of immense importance to understand the factors that may correlate with the formation and stability of perovskite structure type ABO_3_ compounds for the design of new compounds with novel structures containing desired valence states of B cations.

The reason for why many compounds would take perovskite structure type ABX_3_ had been explained from ion-packing & tolerance factor, by Goldschmidt in 1926.^1^ Even now, most of the research articles and text books on the assessment of stability of perovskite structure type ABO_3_ oxides focus on the geometric parameters such as tolerance factor, and structure-field map.^2–5^ However, lattice and valence stabilities should be more related to thermodynamic energy and originate from thermodynamic principles rather than numerical geometrical parameters. Crystal chemistry and thermodynamic energy of ionic solids are reflected into Madelung electrostatic potential as shown by Van Gool and Piken in 1969.^6,7^ “The valence stability using lattice site-potential” of perovskite structure type ABO_3_ was demonstrated by one of the authors of this article (M. Yoshimura) and was considered for the first time in 1974.^8^ This was referred by Rustam Roy in 1975 in a plenary lecture presented as the Robert B. Sosman Memorial Lecture at the 77th annual meeting of the American ceramics society and was published in 1977.^9^ This is a novel and very less explored concept. We also note that not many researchers have been able to consider this concept perhaps due to the inaccessibility of the original article.^8^ Here, the present article is written by re-visiting Madelung lattice energy and site potentials based upon recent data calculated using VESTA^10^ program and recent crystallographic (structural) and thermodynamic data. We emphasize on the fact that ionic solids are stabilized due to the gain in lattice energy that is reflected in Madelung electrostatic potential (*E*_M_). Nevertheless, recently, Hoppe demonstrated that the enthalpy of formation of solids is reflected in Madelung electrostatic potential in 1995^11^ and Glasser has shown that for ionic solids the lattice energy and Madelung electrostatic potential energy (*E*_M_) has a linear relationship in 2012.^12^

**Fig. 1:**
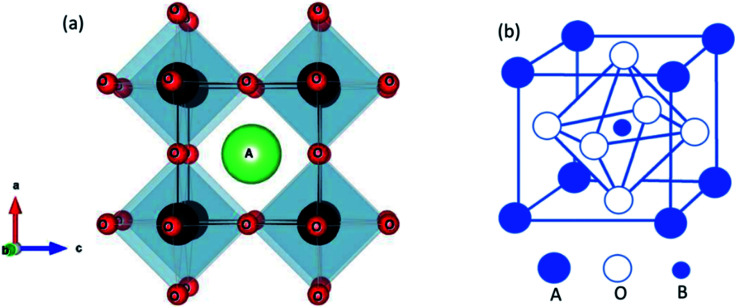
Ideal cubic perovskite structure of ABO_3_ (a) showing corner shared BO_6_ octahedra and A cation at the interstitial position and (b) showing B cation and BO_6_ octahedra at the center of the unit cell.

The ideal perovskite structure type of ABO_3_ compound has a cubic unit cell. This structure can be constructed from corner shared BO_6_ octahedra with A cation being present at the 12 coordinated interstitial position within the octahedral network (Fig. 1a). A and B cations are coordinated by 12 and six O^2−^ ions, respectively and O^2−^ ion is coordinated by four A and two B cations. Several structural modification of cubic perovskite structure has been identified such as tetragonal, orthorhombic, rhombohedral, hexagonal, and monoclinic, respectively either as polymorph of ABO_3_ or upon chemical modification. The most important chemical fact of ABO_3_ perovskite compound is that, apart from noble gasses and nonmetals, A or B in ABO_3_ can be chosen from almost all elements and several aliovalent A or B ions such as 
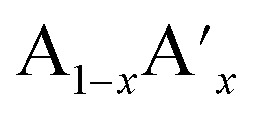
 or 
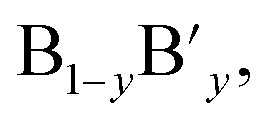
 respectively can be combined in a single compound. Furthermore, non-ideal stoichiometry of oxygen or A such as ABO_3−*z*_ or A_1−*x*_BO_3_, respectively are regularly observed. It is further interesting that many BO_3_ compounds can adopt perovskite type structure where A cation is completely absent for example ReO_3_. Therefore, the structural skeleton formation by BO_3_ sub lattice is generally regarded as prerequisite for the formation of perovskite structure type compounds.^2^ Furthermore, the BO_3_ sub lattice skeleton is primarily stabilized by the electrostatic potential energy gain by ionic arrangements in solid phase and additional stability of perovskite type ABO_3_ compounds can arise from (i) the presence of large A cation within the interstitial position, (ii) crystal field splitting and (iii) hybridization of orbitals.^2^

The total internal energy of ionic solids may be expressed as

1 *E* = *E*_M_ + *E*_N_

where *E*_M_ is Madelung electrostatic potential energy and *E*_N_ is combination of all other energies (repulsive, zero point, crystal field stabilization, vibrational, van der Waals and covalent, energies respectively).^13^

The analysis of Madelung electrostatic potential and lattice site potential of a large number ABO_3_ compounds (with varying combination of A and B) led to a set of fundamental criteria for the formation of perovskite structure type ABO_3_ with varying combination and valence states of A and B and summarized by Yoshimura *et al.*^8,14^ as follows: oxides of A can be reacted with oxides of B to form perovskite structure type ABO_3_ compounds if: (i) B ion is smaller than A ion, (ii) valence of B ion is larger or equal to A ion and (iii) sum of valence of A and B ion is six (including mixed valence situation 
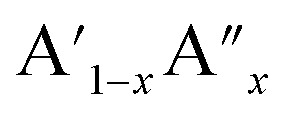
 or 
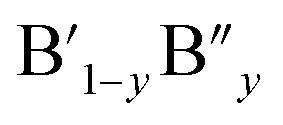
). An in depth analysis showed that the valence stability originates from gain of lattice site potential. Recently, there have been some excellent research work reported in the literature based on the use of Madelung electrostatic potentials energy for understanding the stability of perovskite structure type compounds. Shan *et al.* investigated Li ion insertion in perovskite structure type SrVO_3−*δ*_, La_2/3_TiO_3−*δ*_ and (La, Li)TiO_3−*δ*_, from the lattice site potential analysis and found that the insertion of lithium ion is limited by the effect of lattice site potential on the valence stability of V and Ti.^15^ In these perovskite structure type oxides, the valence state of tetravalent V and Ti can be reduced to 3 upon insertion of monovalent Li ion at an interstitial site. It is interesting to note that for spinel structure type LiMn_2_O_4_ compounds, Ragavendran *et al.* have shown that the Madelung electrostatic potential of Li_*x*_Mn_2_O_4_ almost linearly correlates with *x* and therefore voltage of Li-ion battery made using LiMn_2_O_4_ can be easily estimated from the Madelung electrostatic potential.^16^ Furthermore, the perovskite structure-type is not limited to ABO_3_ and a large number of organic–inorganic halide crystallise into perovskite type structure with general formula ABX_3_. A bottle neck for the realization of full potential of so called ‘perovskite solar cells’ and related electronic devices has been the degradation of electronic devices fabricated using hybrid organic–inorganic ABX_3_ compounds.^17^ The degradation of these devices arise from instability of the perovskite structure type hybrid compounds and can also be understood from Madelung electrostatic potential analysis.^18^ Furthermore, it has also been shown that the stability of oxides such as Li_4_SiO_4_ towards the reaction Li_4_SiO_4_ + CO_2_ → Li_2_SiO_3_ + Li_2_CO_3_ has direct consequence on the CO_2_ capture and storage abilities and recent study by Oh-ishi *et al.* had successfully employed lattice site potentials analysis of all Li^+^ ions in the unit cells of Li_4_SiO_4_, Li_2_SiO_3_, and Li_2_CO_3_ to evaluate the extraordinary stability.^19^

On the other hand, lattice site potential can be used for the estimation of the oxygen to metal charge transfer and bandgap of perovskite type ABO_3_ oxides using an ionic model developed by Zaanen *et al.*^20^ and demonstrated in a large number of oxides by Torrance *et al.*^21^ and Arima *et al.*^22^ Kato *et al.* employed lattice site potential analysis to show that the valence band structure can be tailored by designing the stacking sequence of layers of a number of layered compounds BiOX (X = Cl, Br, I), Bi_4_NbO_8_X (X = Cl, Br), Bi_2_GdO_4_X (X = Cl, Br), and SrBiO_2_X (X = Cl, Br, I) and therefore, lattice site potential analysis may be useful for designing new photocatalysts.^23^ Here we look back at the development of Madelung electrostatic potential and lattice site potential only for perovskite type ABO_3_ oxides. We show that the lattice and valence stabilities of perovskite structure type ABO_3_ compounds can be understood from simple Madelung electrostatic potential view point and valence stability is directly related to the lattice site potential. Based on the existing literatures, our hypothesis is that the stability and properties of perovskite type ABO_3_ oxides can be rationalized better in terms of change in Madelung electrostatic potential energies rather than structural perturbation such as distortions and tolerance factors.^24,25^

The Madelung electrostatic potential and lattice site potentials can be calculated using commercial first principle calculation software packages. We have used freely available software VESTA^10^ to recalculate the lattice site potentials and Madelung electrostatic potential directly from the known crystal structures. The Madelung electrostatic potential *E*_M_ obtained from VESTA has the unit of MJ mol^−1^ asymmetric unit. In order to compare *E*_M_ per mole of each compound with different crystal symmetry (space groups), the *E*_M_ output of each compound from VESTA was multiplied by the multiplicity of general Wyckoff position and divided by the number of formula units in the unit cell.

## Results and discussion

According to Van Gool and Piken,^6,7^ the Madelung electrostatic potential energy *E*_M_ can be expressed as2
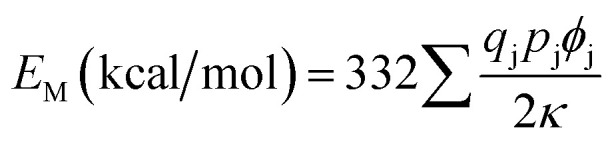
where, *q*_j_ is total charge number of lattice point *j*, *p*_j_ is frequency of occurrence of *j* lattice point in the unit cell, *ϕ*_j_ is the lattice site potential (Å^−1^) at the lattice point *j*, and *κ* is total number of molecules in the unit cell.

The Madelung constant can be expressed as3
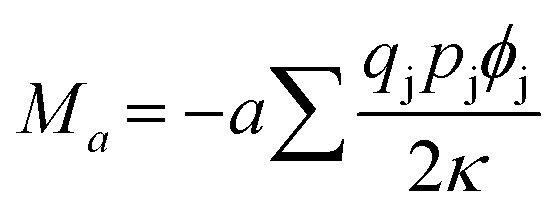
where *M*_*a*_ is expressed in terms of the characteristic length *a*, such as the lattice parameter of unit cell. For compounds with complex structures an arbitrary characteristic length *r* such as nearest neighbor atomic distance can be defined and the Madelung constant can be expressed as4
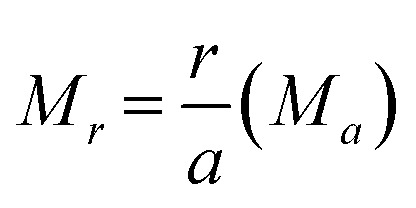


From eqn (2)–(4) the Madelung electrostatic potential can be expressed by5
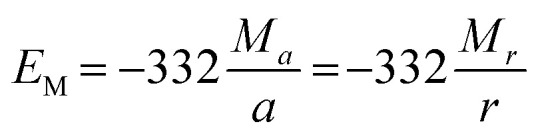


In VESTA,^10^ lattice site potential *ϕ*_i_ and Madelung electrostatic potential *E*_M_ of a given structure is calculated using following equations6
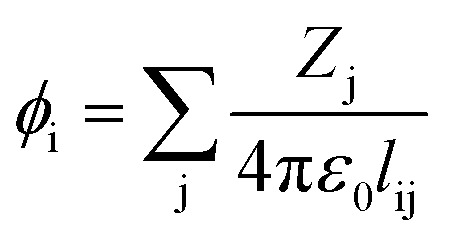
where *Z*_j_ is valence of ion j, *ε*_0_ is permittivity of vacuum and *l*_ij_ is distance between ions i and j.7
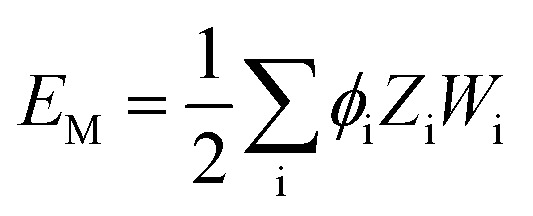
where 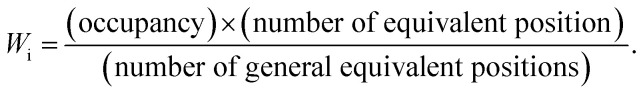


**Fig. 2:**
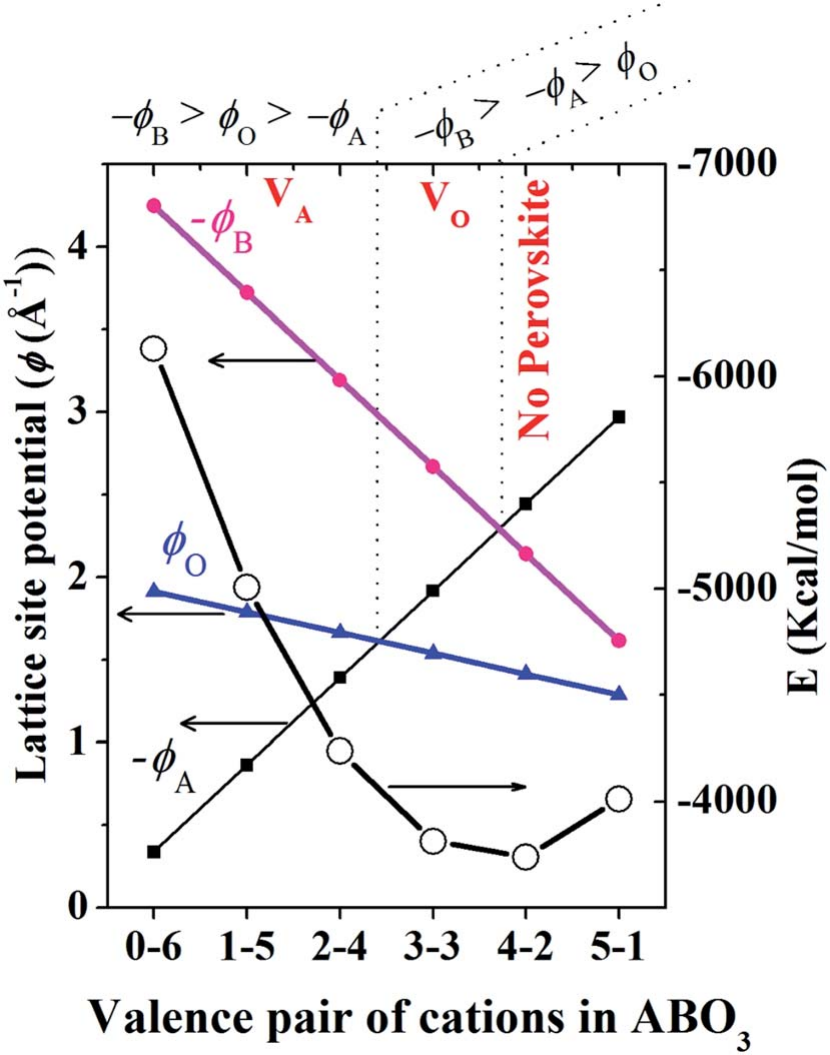
Variation of lattice site potential and Madelung electrostatic potential energy with varying valence-pair compositions of valences of A and B of perovskite structure type ABO_3_ oxides. It should be noted that the sign of *ϕ*_A_ and *ϕ*_B_ are negative. It was assumed that all the valence pairs have ideal cubic perovskite structure (*a* = *b* = *c* = 3.881 Å and *α* = *β* = *γ* = 90° and space group *Pm*3̄*m* (221)).

For similar structures with fixed atomic position (*e.g.* NaCl, CsCl), the lattice site potential *ϕ*_j_ for each site in the crystal structure is inversely proportional to the characteristic length. However, if the characteristic length is fixed, for the first time Yoshimura *et al.*^8^ had analyzed that, lattice site potential *ϕ*_j_, the Madelung constant *M*_a_ and Madelung electrostatic potential energy *E*_M_ varies with the valence states. Fig. 2 shows the relationship between valence state and lattice site potentials *ϕ*_j_ as well as Madelung electrostatic potential *E*_M_ in ideal cubic perovskite type structure of ABO_3_ with fixed lattice constant of 3.881 Å. Based on eqn (5) the Madelung constant *M*_*a*_ also shows similar trend with valence. Fig. 2 shows that the lattice site potential at A site (*ϕ*_A_) and O site (*ϕ*_O_) increase with the valence (0–6), (1–5), (2–4), (3–3) (the first number indicates valence of A and second number indicates valence of B) while the lattice site potential of B ion (*ϕ*_B_) decreases. This re-calculated trend of lattice potential with valence pairs is in excellent agreement with that obtained from method developed by Van Gool and Piken,^6,7^ and adapted by Yoshimura *et al.* in 1974.^8^ All calculations were performed using VESTA^10^ employing the radius of ionic sphere and reciprocal space range of 1.6 Å and 10 Å^−1^, respectively. For the calculation of *ϕ* and *E*_M_ of A^0^B^6+^O_3_, a dummy A such as Na atom was placed at (½ ½ ½) and B cation Re^6+^ at (0 0 0) atomic coordinates, respectively of space group *Pm*3̄*m* (221) with both valence and occupancy, respectively of A were set to zero. The calculation for valence pairs (4–2) and (5–1) assumed hypothetical A^4+^B^2+^O_3_ and A^5+^B^1+^O_3_ perovskite structure type compounds because they rarely exist.

**Fig. 3:**
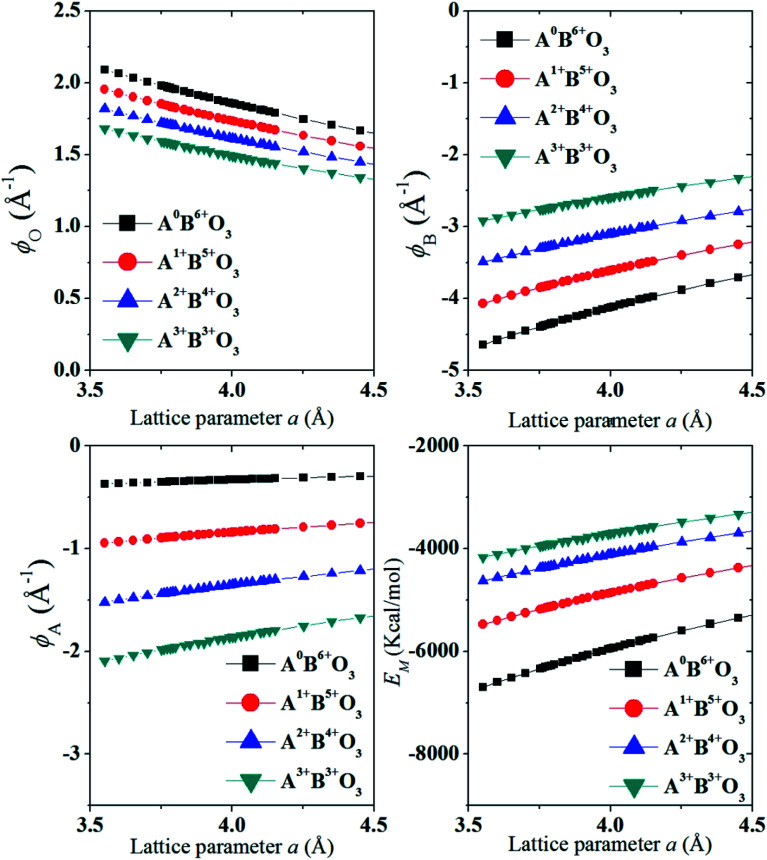
Variation of lattice site potential of different sites *ϕ*_A_, *ϕ*_B_ and *ϕ*_O_ and Madelung electrostatic potential *E*_M_, respectively with lattice parameter of different valence pairs A^0^B^6+^O_3_, (A = Na^0^, B = Re^6+^) as A^1+^B^5+^O_3_, (A = Na^+^, B = Ta^5+^), A^2+^B^4+^O_3_ (A = Sr^2+^, B = Ti^4+^) and A^3+^B^3+^O_3_ (A = La^3+^ and B = Al^3+^) with atomic coordinates of A (½ ½ ½), B (0 0 0) and O (½ 0 0) in space group *Pm*3̄*m* (221). Radius of ionic sphere and reciprocal space range of 1.6 Å and 10 Å^−1^, respectively.

In Fig. 3 we show that, how the lattice site potential (*ϕ*) of each ionic site and Madelung electrostatic potential (*E*_M_) energy changes with lattice parameter when valence is fixed, for each valence pair in each perovskite structure type ABO_3_. The variation observed in Fig. 3 is consistent with the observation of Sabry *et al.* in 2000 (ref. 24). From Fig. 2 and 3 it is observed that lattice site potential for all sites change significantly with valence pairs. Lattice site potential for oxygen in A^3+^B^3+^O_3_ is lowest. Therefore, it can be expected that oxygen can be easily removed from the perovskite type compounds with A^3+^B^3+^O_3_ than A^0+^B^6+^O_3_. Indeed, the occurrence of oxygen vacancy is very common in A^3+^B^3+^O_3_ and this may be of importance during designing catalysts for the availability of active lattice oxygen or designing materials for p- or n- type conductivity.^24,26^

**Scheme 1:**
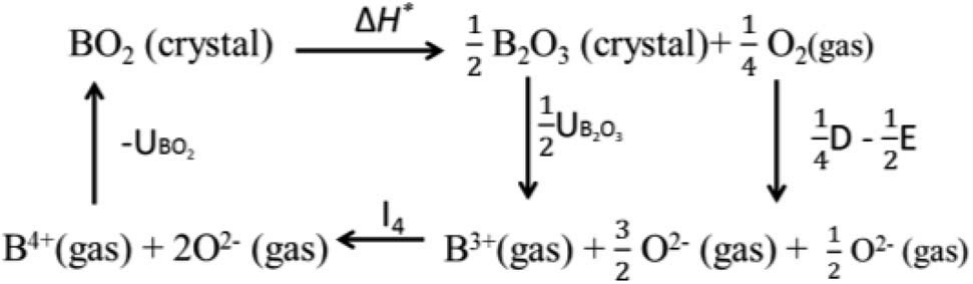
The Born–Haber cycle of dissociation of BO_2_ to B_2_O_3_. Here, UBO_2_ and UB_2_O_3_ are lattice energies of BO_2_ and B_2_O_3_, respectively. I_4_ is ionization energy of B^3+^ to B^4+^. D and E, respectively are dissociation energy and electron affinity of oxygen.

Valence stability is related to lattice energy which mainly arises from Madelung electrostatic potential and more directly related to lattice site potential. We first consider the valence stability of B ions in oxides where both 4+ and 3+ valence state of B ion is known. Example of such B ions are Ce^3+/4+^, Pr^3+/4+^ and Tb^3+/4+^. In order to understand the relative stability of B^4+^O_2_ and B_2_^3+^O_3_ we consider the enthalpy change (8) of the decomposition reaction BO_2_ → ½B_2_O_3_ + ¼O_2_ and corresponding Born–Haber cycle (Scheme 1)

8 ΔH* = (UBO_2_ − ½UB_2_O_3_) − *I*_4_ − ¼*D* + ½*E*

Eqn (8) suggests that high valence of B in oxide BO_2_ can be stable if its lattice energy is compensated by other energy terms. Since, the lattice energy is enthalpy at *T* = 0 K (Δ*H*^o^ = Δ*U*^o^ = Δ*G*^o^; the energy terms such as Δ*S* and RT do not contribute at *T* = 0 K), one can postulate that the stability of high valence ions is enhanced in crystal structure with large lattice energies. A similar analogy can be drawn for the valence stability in complex oxides where multiple ions are present such as in perovskite type ABO_3_ compounds. Furthermore, cations provide large electrostatic potential contribution to the total Madelung electrostatic potential in crystal structure as shown in the plots of *ϕ*_A_ and *ϕ*_B_ in Fig. 3. This is in excellent with the trend of cation site potentials observed by Sabry *et al.*^24^ Therefore, the valence stability of cation is rather directly related to the lattice-site potential of the cation site in a crystal structure and the stability enhances when they are present in lattice site with strong lattice site potential such as B site in perovskite structure type ABO_3_ oxides.

A number of experimental study has indicated that high valence of some B cation is stable in perovskite type A^2+^B^4+^O_3_ oxides but not in fluorite type B^4+^O_2_. For example, investigation on the synthesis of perovskite type ABO_3_ compounds had revealed that several stable perovskite type A^2+^B^4+^O_3_ compounds (with A = Ba, Sr and B = Ce, Tb, Pr) can be synthesized and are stable at room temperatures in spite of the poor ambient condition stability of Pr^4+^O_2_ and Tb^4+^O_2_ and existence of both Ce_2_^3+^O_3_ and Ce^4+^O_2_.^27^ In order to explain this experimental fact, an in depth analysis of lattice site potential and Madelung site potential was presented in.^8^ Here, we have recalculated the lattice site potential and Madelung electrostatic potential using VESTA^10^ and presented in Table 1. Our recently calculated values show excellent matches with that using the procedure of Van Gool and Piken.^8^ In Table 1 the lattice site potential of A ion in all perovskite structure type compounds appear to be close to those of AO but for B ion, the perovskite structure provides significant lattice site potential gain than that in fluorite structure. Except for BaTbO_3_ which we believe due to not so accurate estimation of lattice parameter for TbO_2_.

**Table 1:** Madelung electrostatic potential energy of some different structured oxides, fluorite (AO_2_) rock salt (AO) and perovskite (A^2+^B^4+^O_3_) types. *ϕ*_A_, *ϕ*_*B*_ and *ϕ*_*O*_ are lattice site potentials of A, B and oxygen sites, respectively (see text). The data were calculated using VESTA^10^ employing radius of ionic sphere and reciprocal space range of 1.6 Å and 10 Å^−1^, respectively

Compound	Crystal structure	Lattice constant^a^ (Å)	Madelung electrostatic potential *E*_M_ (kcal mol^−1^)	Difference Δ*E*_M_^b^ (kcal mol^−1^)	Lattice site potential (Å^−1^)		
					*ϕ*_A_	*ϕ*_B_	*ϕ*_O_
CeO_2_	Fluorite	5.411^a^	−2855.431			−2.801	1.504
PrO_2_		5.393^a^	−2864.947			−2.811	1.509
TbO_2_		5.22^a^	−2959.725			−2.904	1.559
BaO	NaCl	5.523^a^	−840.791		−1.269		1.269
SrO		5.160^a^	−899.696		−1.358		1.358
BaCeO_3_	Perovskite	4.396^b^	−3738.779	−42.6	−1.228	−2.822	1.469
BaPrO_3_		4.372^b^	−3759.255	−53.5	−1.234	−2.837	1.477
BaTbO_3_		4.280^b^	−3839.885	−39.4	−1.261	−2.898	1.509
SrCeO_3_		4.291^b^	−3830.060	−74.9	−1.258	−2.890	1.505
SrPrO_3_		4.280^b^	−3839.885	−75.2	−1.261	−2.898	1.509
SrTbO_3_		4.186^b^	−3925.916	−66.5	−1.289	−2.963	1.543

a Pseudocubic lattice parameters from a: ref. 31 and b: ref. 27.

b Δ*E*_M_ = *E*_M_(ABO_3_) − [*E*_M_(AO) + *E*_M_(BO_2_)].

**Fig. 4:**
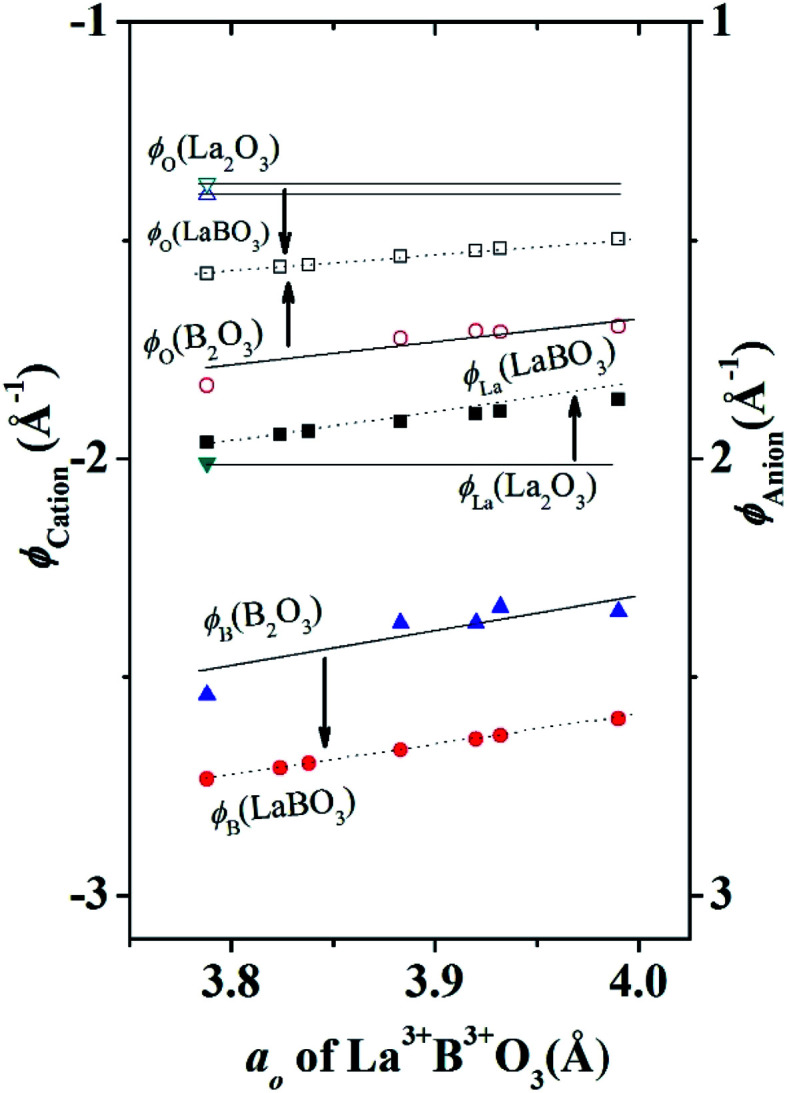
Variation of lattice site potential *ϕ*_La_, *ϕ*_B_ and *ϕ*_O_ of different sites as a function of pseudocubic lattice parameter (*a*_o_) of several LaBO_3_ and compared with lattice site potentials of B_2_O_3_ or La_2_O_3_. Solid symbols are for cations and hollow symbols are for anion, both dot and solid lines are guide to the eyes.

Therefore, the experimental observation that B^4+^ was not stable in fluorite structure type B^4+^O_2_ but stable in perovskite structure type A^2+^B^4+^O_3_ is in accordance with the data presented in Table 1. The strong lattice site potential at B site results in large total Madelung electrostatic potential of perovskite structure. In general, Fig. 4 shows that lattice site potential of B site is stronger than A or O site in perovskite type ABO_3_ in each valence state situation. Therefore, we find that the formation of these perovskite type oxides are associated with significant gain in Madelung electrostatic potential (Δ*E*_M_) calculated by subtracting the combined *E*_M_ of rock salt structure type AO and fluorite structure type BO_2_ from the *E*_M_ of perovskite crystal structure type of final ABO_3_ (Table 1). For example, one can reasonably expect that Ba^2+^O + Ce^4+^O_2_ → Ba^2+^Ce^4+^O_3_ reaction may proceed as there is gain in Madelung electrostatic potential Δ*E*_M_ of ∼ −43 kcal mol^−1^. It should be noted that this discussion of lattice site potential and Madelung electrostatic potential is first approximation but still can be useful to explain experimental observations. For the calculation of the *E*_M_ and *ϕ*, an ideal cubic structure for ABO_3_ has been assumed and all the ions have been considered as point charges. In real crystals however, ions will have certain degree of polarization in their site, and various structural distortion generally leads to the deviation from cubic symmetry of the crystal structure of the perovskites. High structural distortion can lead to the decomposition of ABO_3_ to component oxides. Therefore, the practical stability of these perovskite structure type ABO_3_ oxides may not be very high.

Takayama-Muromachi *et al.* experimentally obtained change in heat of formation (Δ*H*_f_) for the reaction A^2+^O + B^4+^O_2_ → A^2+^B^4+^O_3_ of several oxides (A = Ca, Sr, Ba, Pb and Cd; B =Ti and Zr).^13^ They also calculated change in Madelung electrostatic potential (Δ*E*_M_) from the *E*_M_ of A^2+^B^4+^O_3_, AO and BO_2_ and showed that heat of formation of perovskite structure type compounds can be written as

9 Δ*H*_f_ ≈ Δ*E* = Δ*E*_M_ + Δ*E*_N_

It has also been shown that Δ*E*_M_ correlates well with Goldschmidt tolerance factor 10
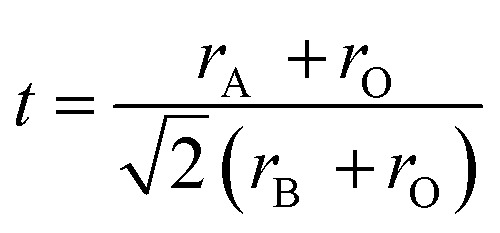
(where *r*_A_, *r*_B_ and *r*_O_ are ionic radius of perovskite structure type ABO_3_) and Δ*E*_M_ becomes more negative when *t* reduces from 1 to 0.8. Kamata *et al.* found that lattice site potential for B and Madelung electrostatic potential of A^2+^B^4+^O_3_ (here A = Ca, Sr and Ba and B = Mo), varies linearly with pseudocubic lattice constant and the lattice site potential of Mo is much deeper in perovskite type structure than MoO_2_.^28^ It is further interesting to note that some of these perovskite-type oxides are so stable that, they are not easily reduced under hydrogen atmosphere at moderate temperatures. From above examples, it can be reasonably hypothesized that the gain in lattice site potential of B site and hence the Madelung electrostatic potential can promote the formation of perovskite structure type ABO_3_ compounds. Furthermore, from the lattice site potential consideration, it can be shown that Ca^2+^Mn^4+^O_3_ and A^2+^Pb^4+^O_3_ (A = Ba, Sr) are stable under conditions where the corresponding MnO_2_ or PbO_2_ decomposes to lower valence oxides.^8^

The valence stabilization due to strong lattice potential at B site as described above for A^2+^B^4+^O_3_ can be further extended to A^3+^B^3+^O_3_. For example, hexagonal structured A_2_O_3_ (such as La_2_O_3_) can be reacted with rhombohedral (corundum) structured B_2_O_3_ (such as Cr_2_O_3_) or rutile structured BO_2_ (such as CrO_2_) to form perovskite structure type A^3+^B^3+^O_3_ compounds. However, high valence Ni^3+^ and Co^3+^ in corundum structure Ni_2_O_3_ and Co_2_O_3_ respectively are not stable but LaNi^3+^O_3_, LaCo^3+^O_3_ are stable. Lattice parameters and atomic coordinates for corundum structure Ni_2_O_3_ and Co_2_O_3_ is unavailable. In this situation the Madelung electrostatic potential and lattice site potential, can be calculated using a different characteristic length *r* = *r*_B_ + *r*_O_ for each known A_2_O_3_, B_2_O_3_, BO, BO_2_, and ABO_3_ using eqn (3)–(5) and graphically estimated for unknown compounds.^8^ Further detailed discussion on the characteristic length and Madelung electrostatic potential can be found in.^8,12^ However, for comparison purposes, here all lattice site potentials were obtained using VESTA^10^ that uses eqn (6) and (7) for lattice site potential and Madelung electrostatic potential and presented in Tables 1 and 2. The lattice site potential obtained from compounds with general formula LaB^3+^O_3_ (B = Al, Co, Cr, Fe Ni, Ti and V) and B_2_^3+^O_3_ (B = Al, Co, Cr, Fe, Ti and V), are given in Table 2. Fig. 4 shows the gain in lattice site potentials of all the sites in La^3+^B^3+^O_3_. It can be clearly seen that there is significant enhancement of lattice site potential for B site for all La^3+^B^3+^O_3_ from that of corundum structure B_2_^3+^O_3_. Thus, high valence state of Ni and Co is stable in La^3+^B^3+^O_3_ is due to this large lattice site potential at B site and consistent with similar high valence stability situation of A^2+^B^4+^O_3_ as discussed in the previous section. Furthermore, from Fig. 4 it can be stated that the lattice site potential for oxygen is much lower in perovskite structure than corundum structure and therefore, corundum structure of B_2_O_3_ is more difficult to form oxygen related point defects than perovskite structure type La^3+^B^3+^O_3_. Indeed, a similar analysis has been performed for rutile BO_2_ structure as-well^8^ and therefore it may be understandable that why oxygen vacancy is rarely seen in rutile or corundum structure although plenty of vacancy present in perovskite structure type A^3+^B^3+^O_3_ compounds. Therefore, lattice site potential analysis provides reasonable understanding of the defect formation in perovskite structure ABO_3_ compounds and preparation of many perovskite structure type ABO_3_ phases from constituent binary oxides could be understood due to the strong B site potential and gain of Madelung electrostatic potential Δ*E*_M_.

**Table 2:** Lattice site potentials and Madelung electrostatic potentials of LaB^3+^O_3_, and B_2_^3+^O_3_ compounds calculated using VESTA^10^^a^

Compound	Structure	Lattice parameter (Å)	Ref^b^	Madelung electrostatic potential *E*_M_ (kcal mol^−1^)	Difference Δ*E*_M_ (kcal mol^−1^)	Lattice site potential (Å^−1^)		
						*ϕ*_A_	*ϕ*_B_	*ϕ*_O_
La_2_O_3_	Hexagonal	*a* = *b* = 3.938, *c* = 6.180, *α* = *β* = 90°, *γ* = 120°	i	−3381		−2.0095		1.3945, 1.3687
Al_2_O_3_	Rhombohedral	*a* = *b* = 4.761, *c* = 12.996, *α* = *β* = 90°, *γ* = 120°	ii	−4352			−2.5402	1.8320
Cr_2_O_3_		*a* = *b* = 5.07, *c* = 13.872, *α* = *β* = 90°, *γ* = 120°	iii	−4081			−2.3757	1.7241
Fe_2_O_3_		*a* = *b* = 5.105, *c* = 13.913, *α* = *β* = 90°, *γ* = 120°	iv	−4064			−2.3754	1.7070
Ti_2_O_3_		*a* = *b* = 5.113, *c* = 13.984, *α* = *β* = 90°, *γ* = 120°	v	−4031			−2.3392	1.7097
V_2_O_3_		*a* = *b* = 5.126, *c* = 14.134, *α* = *β* = 90°, *γ* = 120°	vi	−4030			−2.3510	1.6972
LaAlO_3_	Pseudocubic	3.788	vii	−3904	−37	−1.9630	−2.7337	1.5765
LaCoO_3_		3.824		−3867	—	−1.9446	−2.7081	1.5616
LaNiO_3_		3.838		−3853	—	−1.9375	−2.6982	1.5559
LaCrO_3_		3.883		−3808	−77	−1.9152	−2.6670	1.5379
LaTiO_3_		3.92		−3772	−67	−1.8971	−2.6419	1.5234
LaFeO_3_		3.932		−3761	−39	−1.8914	−2.6339	1.5188
LaVO_3_		3.99		−3706	−1	−1.8640	−2.5957	1.4967

a *ϕ*_A_, *ϕ*_B,_ and *ϕ*_O_ are lattice site potentials for A, B and oxygen sites, respectively.

b References for lattice parameters (i) mp-1968; (ii) COD1000032; (iii) mp-19399; (iv) mp-19770; (v) mp-458; (vi) mp-18937; (vii) F. S. Galasso ‘‘Structure, properties and preparation of perovskite type compounds'’, Pergamon Press, New York (1969); mp stands for materials project, and COD stands for crystallography open database.

The perovskite structure type compound A^4+^B^2+^O_3_ with valence pair (4–2) cannot be prepared and rarely exist. This can be rationalized from Madelung electrostatic potential view point as well. For example, the calculation of Δ*E*_M_ of a hypothetical chemical reaction ThO_2_ + MgO → Th^4+^Mg^2+^O_3_ revealed that a net Madelung electrostatic potential loss of 184 kcal mol^−1^ (calculated assuming lattice the parameter of ThMgO_3_ as *a* = 4.008 Å, equivalent to CaZrO_3_ due to similarity of ionic radius of Ca^2+^ and Th^4+^ as well as Mg^2+^ and Zr^4+^ since the lattice parameter of ThMgO_3_ is not available) would occur for the formation of perovskite structure type Th^4+^Mg^2+^O_3_.^14^ Thus this reaction will not proceed because there is no Madelung electrostatic potential or lattice energy gain in this perovskite type A^4+^B^2+^O_3_ compound and the same hypothesis is applicable to A^5+^B^1+^O_3_. We also note that very recently A^4+^B^2+^O_3_ compounds Bi^4+^Ni^2+^O_3_ and Pb^4+^Ni^2+^O_3_ have been reported to be made only under very high pressure conditions and these can be considered as very special valence combinations where stability is perhaps favored due to other energy factors such as covalency due to hybridization.^29,30^

**Scheme 2:**
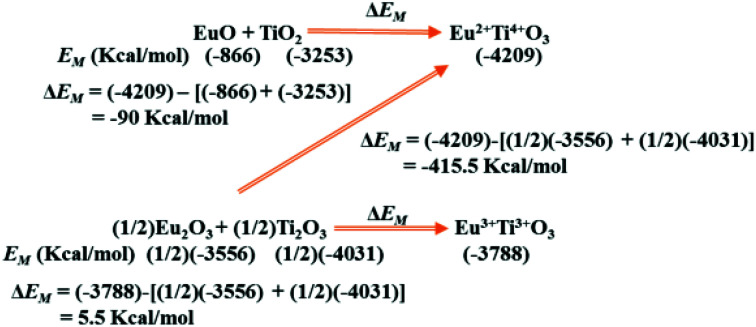
Change in Madelung electrostatic potential Δ*E*_M_ for the formation of EuTiO_3_. The *E*_M_ were calculated using similar conditions as described for other systems using VESTA,^10^ assuming perovskite type Eu^3+^Ti^3+^O_3_ and Eu^2+^Ti^4+^O_3_ may have same lattice parameter of 3.904 Å (as the lattice parameter of Eu^3+^Ti^3+^O_3_ is not available).

In Scheme 2 we show similar (see Table 2) calculation of the difference in Madelung electrostatic potential Δ*E*_M_ in the preparation of perovskite structure type EuTiO_3_ from EuO or Eu_2_O_3_ and TiO_2_ or Ti_2_O_3_. The calculated Δ*E*_M_ in Scheme 2 suggests that Eu^2+^Ti^4+^O_3_ will be more stable than Eu^3+^Ti^3+^O_3_ and usually any preparation will lead to the formation of Eu^2+^Ti^4+^O_3_. This analysis may be expanded to other compounds which are of importance for stabilizing Eu^2+^ at specific ionic sites for the development of visible light emitting phosphor materials.

In conclusion, we have revisited some fundamental aspects of synthesis, stability and properties of perovskite structure type ABO_3_. With the use of lattice site potential and Madelung electrostatic potential we have been able to demonstrate that (1) perovskite structure type ABO_3_ compounds can be synthesized from their parent binary oxides because of gain in Madelung electrostatic potential which directly correlates with lattice energy, (2) Madelung electrostatic potential and lattice site potentials dictates the stability of perovskite structure and valence stability of cation in perovskite structure is direct consequence of strong cation lattice site potential and (3) the formation of defects such as oxygen vacancy or excess is driven by intrinsic lower lattice site potential of oxygen site. These results (1)–(3) can be seen various experimental data^13,32–34^ however, have not been explained in terms of lattice site potential by the authors. Thus, the present article will contribute to understanding of synthesis, structures and properties of functional perovskite type oxides for various areas of applications.

## Conflicts of interest

There are no conflicts to declare.

## Supplementary Material
